# Organic Anion Transporting Polypeptide 1B1 Is a Potential Reporter for Dual MR and Optical Imaging

**DOI:** 10.3390/ijms22168797

**Published:** 2021-08-16

**Authors:** Yi-Hsueh Lee, Menq-Rong Wu, Jong-Kai Hsiao

**Affiliations:** 1 Department of Medical Imaging, Taipei TzuChi General Hospital, Buddhist Tzu-Chi Medical Foundation, New Taipei City 23142, Taiwan; 822leeyh@gmail.com (Y.-H.L.); d04548006@ntu.edu.tw (M.-R.W.); 2 School of Medicine, Tzu Chi University, Hualien 97004, Taiwan

**Keywords:** organic anion transporting polypeptide, near-infrared, reporter gene, Gd-BOTPA, indocyanine green

## Abstract

Membrane proteins responsible for transporting magnetic resonance (MR) and fluorescent contrast agents are of particular importance because they are potential reporter proteins in noninvasive molecular imaging. Gadobenate dimeglumine (Gd-BOPTA), a liver-specific MR contrast agent, has been used globally for more than 10 years. However, the corresponding molecular transportation mechanism has not been validated. We previously reported that the organic anion transporting polypeptide (OATP) 1B3 has an uptake capability for both MR agents (Gd-EOB-DTPA) and indocyanine green (ICG), a clinically available near-infrared (NIR) fluorescent dye. This study further evaluated OATP1B1, another polypeptide of the OATP family, to determine its reporter capability. In the OATP1B1 transfected 293T transient expression model, both Gd-BOPTA and Gd-EOB-DTPA uptake were confirmed through 1.5 T MR imaging. In the constant OAPT1B1 and OATP1B3 expression model in the HT-1080 cell line, both HT-1080-OAPT1B1 and HT-1080-OATP1B3 were observed to ingest Gd-BOPTA and Gd-EOB-DTPA. Lastly, we validated the ICG uptake capability of both OATP1B1 and OATP1B3. OAPT1B3 exhibited a superior ICG uptake capability to that of OAPT1B1. We conclude that OATP1B1 is a potential reporter for dual MR and NIR fluorescent molecular imaging, especially in conjunction with Gd-BOPTA.

## 1. Introduction

Molecular imaging, which involves observing molecular events by using a reporter, has facilitated drug development. Several magnetic resonance (MR) reporters that transport gadolinium or iron oxide–based nanoparticles have been developed [1,2]. The development of MR reporters is crucial for clinical imaging because, unlike fluorescent imaging, MR imaging has no tissue depth detection limit. Like key and lock, MR reporters are specific to a contrast agent; therefore, development of new reporters is influenced by the clinical evolution and advancement of these agents.

Gadolinium-based contrast agents (GBCAs) are widely used in MRI. The gadolinium belongs to the lanthanide series of the periodic table. When gadolinium is oxidized (Gd^3+^), it is paramagnetic and can alter the resonance frequencies of surrounding water molecules, and is capable of differentiating normal and abnormal tissue [3]. However, the Gd^3+^ is toxic to mammals [4]. Chelating Gd^3+^ is essential to prevent toxic effects and to accelerate gadolinium elimination. The most well-known gadolinium chelates are Gadopentetic acid (Gd-DTPA). Some of the DTPA derivatives have also been launched in the clinical field, such as gadobenate dimeglumine (Gd-BOPTA) and Gadoxetate Disodium (Gd-EOB-DTPA). The pharmacodynamics and pharmacokinetics of the GBCAs are different according to the chelating agent used. The GBCAs are divided into either non-organ specific or -organ-specific, depending on which cell membrane transporters are responsible for intracellular uptake. Liver-specific contrast agents such as Gd-EOB-DTPA (trade name: Primovist) and Gd-BOTPA (trade name: MultiHance) are two GBCAs commonly used in molecular imaging [5,6]. Reporters that utilize Gd-EOB-DTPA, specifically apical sodium-dependent bile acid cotransporter (ASBT), sodium taurocholate cotransporting polypeptide (NTCP), and organic anion transporting polypeptide (OATP), were studied to determine their efficacy as MR reporters [7,8]. Although Gd-BOPTA entered clinical use before Gd-EOB-DTPA, the intracellular uptake mechanism of Gd-BOPTA is unknown, hindering its application in molecular imaging.

**Figure 1:**
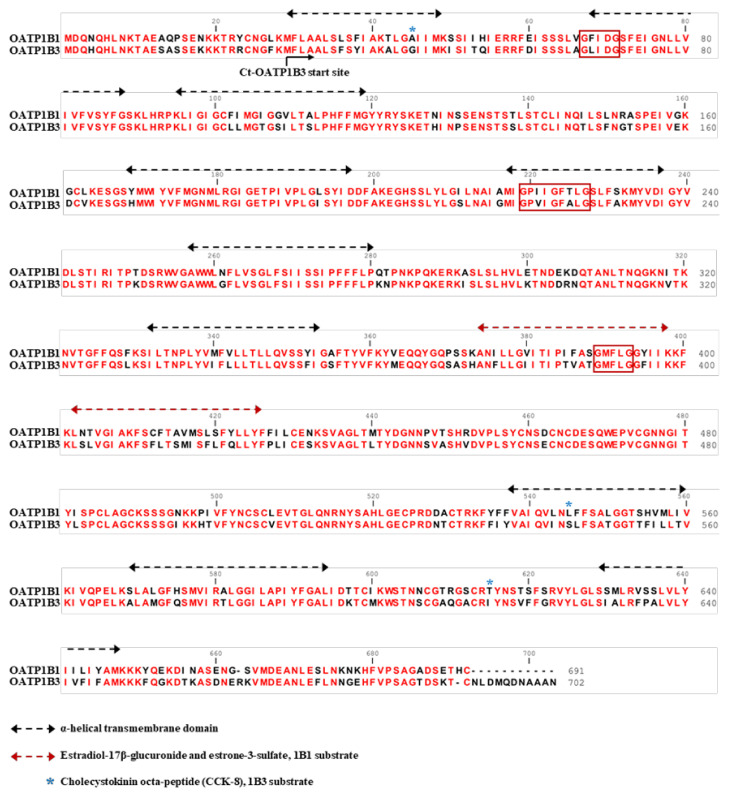
Sequence alignment of human OATP1B1 and OATP1B3. Twelve putative transmembrane domains are under the black dotted lines. The specific domains critical for transport specificity are under the red dotted lines. The amino acid residues the are presumably important for CCK-8 are labeled with a blue star. The conserved amino acid residues between OATP1B1 and OATP1B3 are in red. Three GxxxG motifs associated with oligomerization are boxed in red.

OATPs are 12 transmembrane glycoproteins in the solute carrier organic ion transporter (SLCO) family and are responsible for the cellular uptake of small anion molecules including nutritional molecules in the small intestine and bilirubin in the liver [9]. In humans, 11 OATPs have been identified. OATP1B1 and OATP1B3 account for a high proportion of transporter proteins in the liver [10]. As shown in Figure 1, despite OATP1B1 and OATP1B3 both being liver-specific reporters and sharing 80% of their amino acid sequences [11,12,13,14], studies have shown that they have divergent substrate specificity and preferences. Estradiol-17β-glucuronide and estrone-3-sulfate are two substrates that are transported preferentially by OATP1B1, whereas cholecystokinin octa-peptide (CCK-8) is a substrate whose transport is specific to OATP1B3 [11,12,13,14]. Previous inhibition studies and activity modification via the protein kinase C pathway showed that Gd-BOPTA is transported through OATPs and that its efflux from hepatocytes is via multiple resistance-associated protein 3 [15,16]. However, other results identified human OATP1B1 and OATP1B3 expression in *Slco1a/1b* knockout mice and that OATP1B1/1B3 could not fully rescue the transportation of Gd-BOPTA in knockout mice [17]. According to these studies, the OATP family transportability toward Gd-BOPTA is not determined and we intend to use a cell line transduction model to investigate the Gd-BOPTA transport capabilities of human OATP1B1 and OATP1B3 separately.

ICG is a near-infrared fluorescent (NIR) dye used clinically for evaluating liver function and determining retinal vasculature [18]. Due to its NIR fluorescent character, which is advantageous in deep tissue detection, ICG has been proposed to be a molecular imaging contrast agent [8]. We previously verified that ICG could be ingested by membrane proteins such as OATP1B3, NTCP, and ASBT. However, little is known regarding the intracellular uptake of ICG by OATP1B1. In our previous study, OATP1B3 was determined to be a dual-imaging reporter because it was found to transport ICG and Gd-EOB-DTPA both in vitro and in vivo [2].

In this study, we discovered the capabilities of OATP1B1 acting as a reporter for Gd-BOPTA and ICG in the cell model. The primary goal was to verify the transportation of Gd-BOPTA and ICG by OATP1B1. The secondary objective was to compare the MR reporter capabilities of OATP1B1 and OATP1B3 by using a 1.5 T clinical MR imaging (MRI) system.

## 2. Results

### 2.1. Gd-BOPTA and Gd-EOB-DTPA Uptake by Transient OATP1B1-Expressing HEK293T Cell Line

**Figure 2:**
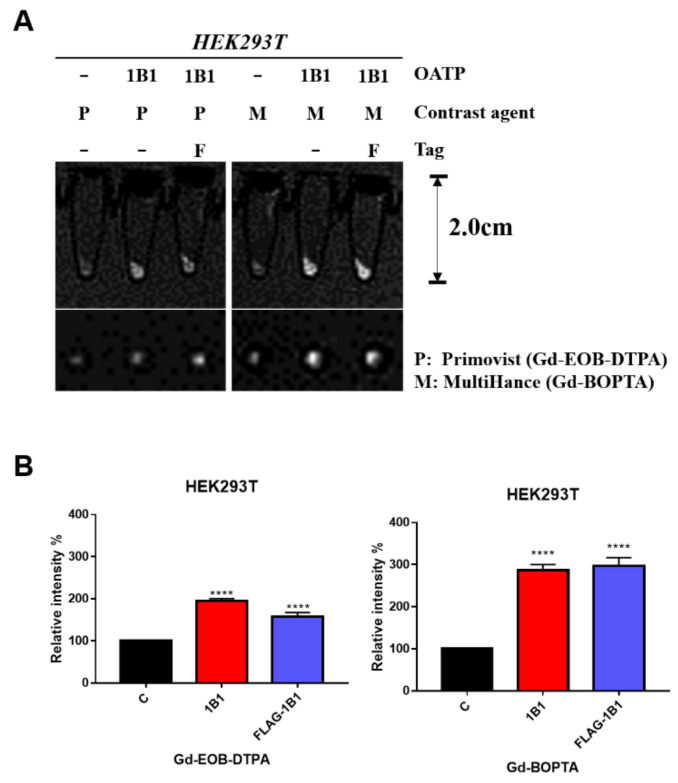
In vitro MRI contrast agent uptake in 293T cells. (**A**) OATP-expressing and untransfected 293T cells were treated with MRI contrast agents. After washing with PBS three times, the cells were scanned with a 1.5-T MRI system. (**B**) The quantitative signal intensity of cell MRI (*n* = 3). Error bars show the standard error and mean, and **** represents results with *p* < 0.0001. C, control; 1B1, OATP1B1-expressing cells; F, OATP1B1 fused with FLAG tag; P, Primovist (Gd-EOB-DTPA); M, MultiHance (Gd-BOPTA).

To investigate OATP1B1 transportation of Gd-EOB-DTPA, we used 293T cells to transiently overexpress OATP1B1 and measured the uptake efficiency of Gd-BOPTA and Gd-EOB-DTPA by imaging with a 1.5-T MRI system. In addition, OATP1B1 proteins with and without the N-terminal FLAG tag were separately transfected into the 293T cells, and the effects of this tag on transportation were investigated. As shown in Figure 2, both OATP1B1- and FLAG-OATP1B1-expressing 293T cells had increased Gd-EOB-DTPA signal intensity in cellular MRI. The enhancement of the MR signal was slightly higher in OATP1B1-expressing cells than in FLAG-OATP1B1-expressing cells upon visual inspection (Figure 2A). Moreover, both OATP1B1- and FLAG-OATP1B1-expressing 293T cells had higher signal intensity enhanced by Gd-BOPTA. Further quantitative analysis indicated that the Gd-EOB-DTPA signal intensity increased by 1.5–2 times and the Gd-BOPTA signal intensity increased by approximately three times in comparison with untransfected 293T cells (Figure 2B), suggesting that OATP1B1 is an efficient reporter for Gd-BOPTA and Gd-EOB-DTPA.

### 2.2. Validation of SLCO1B1 Gene Construction and mRNA Expression

**Figure 3:**
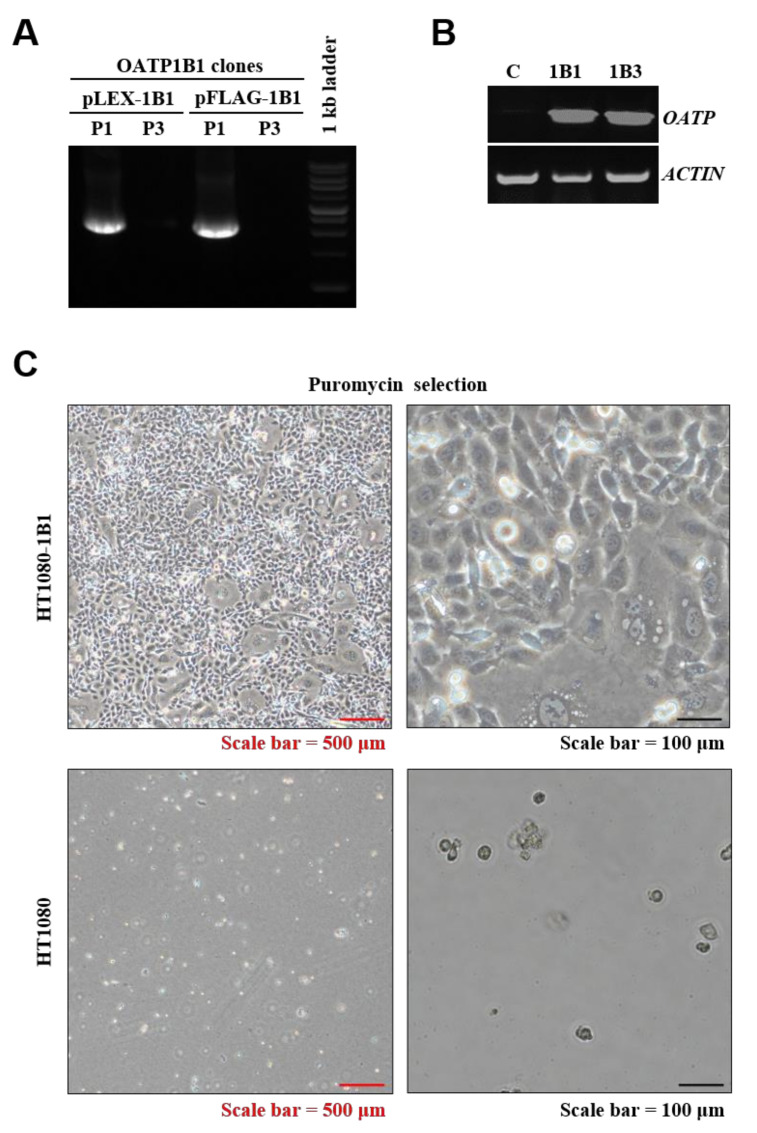
Validation of OATP1B1 construction and stable-expressing cell line. (**A**) PCR showing insert fragments of OATP1B1 (2037 bp). (**B**) RT-PCR revealing the mRNA expression level of OATP1B1- and OATP1B3-expressing cells. (**C**) Morphology of OATP1B3-expressing HT1080 and untransduced HT1080 under 2 μg/mL puromycin selection. P1, OATP1B1-specific primer; P3, OATP1B3-specific primer; C, control; 1B1, OATP1B1-expressing cells; 1B3, OATP1B3-expressing cells.

To construct OATP1B1, which is encoded by *SLCO1B1* (Gene ID: 10599), an open reading frame (2073 bps) driven by a cytomegalovirus promoter was cloned separately into lentiviral pLEX-MCS and pFLAG vectors. Specific polymerase chain reaction (PCR) primers verified positive plasmids. The results were compared with our OATP1B3 clones, which were constructed in previous studies [2]. As shown in Figure 3A, PCR products appeared nearly 2 kb in size after being amplified by an OATP1B1-specific primer. The results of a PCR with an OATP1B3-specific primer indicated an absence of non-specific amplified products. Finally, the positive clones were named pLEX-1B1 and pFLAG-1B1.

Next, the pLEX-1B1 plasmid was transduced into HT1080 cells to generate an OATP1B1-expressing stable cell line. After puromycin selection for 14 d, mRNA expression of OATP1B1 was confirmed using reverse-transcription PCR, and the results were compared with our OATP1B3-expressing cell line, which was produced in previous studies. As shown in Figure 3B, the target gene expression level of OATP1B1-expressing cells is similar to that of OATP1B3-expressing cells. The morphology of OATP1B1-expressing cells was observed during selection, and most OATP1B1-expressing cells were able to tolerate the stress from puromycin. The untransduced cells indicated that a kill dose (2 μg/mL) of puromycin was efficient for HT1080 cell selection (Figure 3C).

### 2.3. Detection of OATP1B1 Protein Expression in HT1080 Cell Line

**Figure 4:**
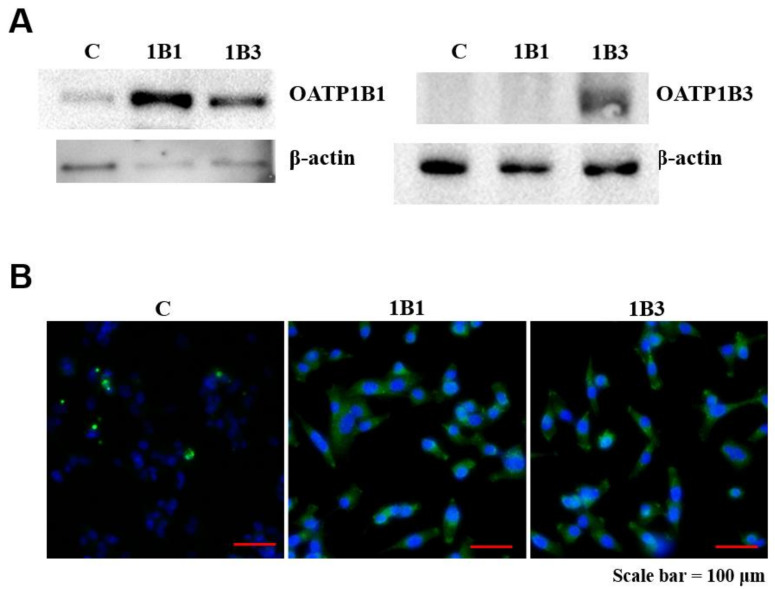
Protein expression of OATP1B1 and OATP1B3. (**A**) Western blot analysis of OATP1B1, OATP1B3, and control β-actin for OATP1B1-expressing, OATP1B3-expressing, and control cells. (**B**) Fluorescence microscopy displaying staining for OATP1B1, OATP1B3 (green), and nuclei control (blue). C, control; 1B1, OATP1B1-expressing cells; 1B3, OATP1B3-expressing cells.

The protein expression of the OATP1B1-expressing HT1080 cells was confirmed through both Western blotting and immunofluorescence methods. In Western blotting, OATP1B1- or OATP1B3-expressing HT1080 cells had a detectable level of the OATP1B1 or OATP1B3 protein compared with empty HT1080 cells (Figure 4A). The AlexaFluor 488 antibody was used to stimulate HT-1080 cell immunofluorescence and visualize OATP1B1 or OATP1B3 expression, which was mostly in OATP1B1-transduced or OATP1B3-transduced cells. OATP1B1 and OATP1B3 were identified in both the cytoplasm and the cell membrane (Figure 4B).

### 2.4. Validation and Comparison of Gd-BOPTA and Gd-EOB-DTPA Uptake of OATP1B1- and OATP1B3-Expressing HT1080 Cell Lines

**Figure 5:**
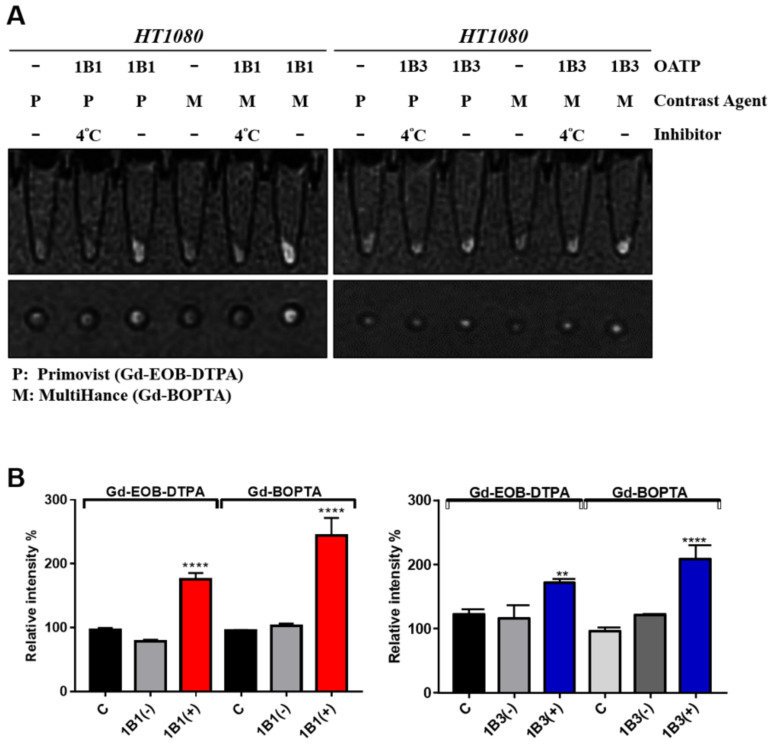
In vitro uptake of MRI contrast agents in HT1080. (**A**) OATP-expressing and untransduced HT1080 cells were treated with MRI contrast agents. The inhibitor group was incubated at 4 °C. After PBS washing three times, cells were scanned with a 1.5-T MRI system. (**B**) The quantitative signal intensity of cell MRI (*n* = 3). Error bars show the SEM, and ** and **** represent *p* < 0.01 and *p* < 0.0001, respectively. C, control; 1B1, OATP1B1-expressing cells; (−), inhibitor group; (+), normal group; P, Primovist; M, MultiHance.

The Gd-EOB-DTPA and Gd-BOPTA signal intensity of the OATP1B1-expressing cells was higher than that of the control cells visually and was reduced through incubation at 4 °C (Figure 5A, left panel). A similar result was observed for OATP1B3-expressing cells (Figure 5A, right panel). The quantitative data indicated that MR signal intensity was increased by 1.8–2.5 times compared with the control cells (Figure 5B). Moreover, the OATP1B1-expressing cells had better transportation efficiency for Gd-BOPTA than for Gd-EOB-DTPA. Unexpectedly, the OATP1B3-expressing cells also increased the MR signals of both Gd-BOPTA and Gd-EOB-DTPA (1.7 and 2 times, respectively), implying that both OATP1B1 and OATP1B3 are capable of reporting the MR signals of both Gd-BOPTA and Gd-EOB-DTPA.

### 2.5. In Vitro ICG Uptake of OATP1B1- and OATP1B3-Expressing HT1080 Cell Line

**Figure 6:**
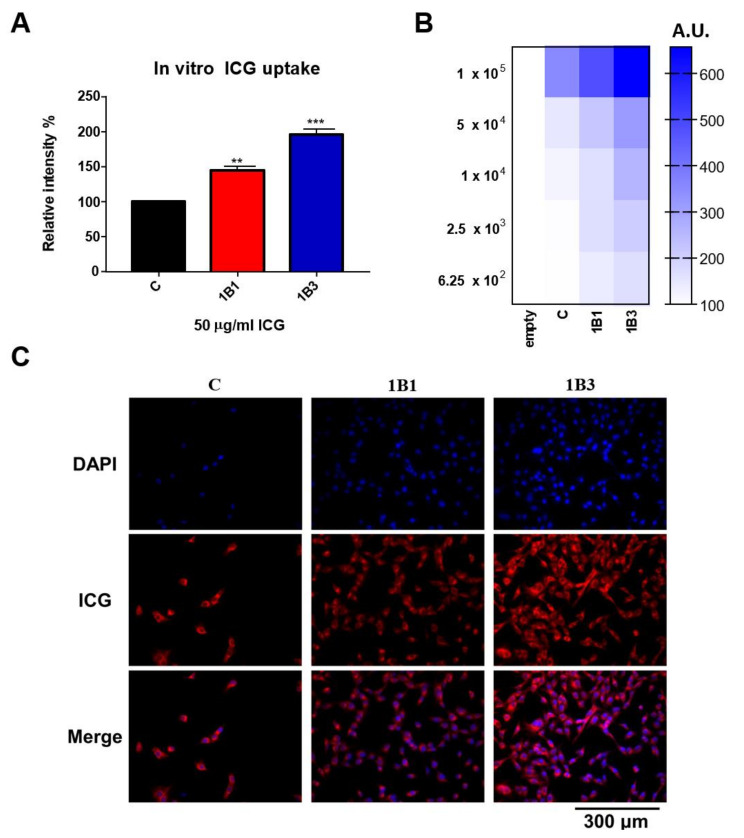
In vitro ICG uptake in HT1080 cells. (**A**) OATP-expressing and untransduced HT1080 cells were treated with 50 μg/mL ICG for 1 h. After washing with PBS three times, the signals were detected with a fluorescence reader (*n* = 3). Error bars show the SEM, and ** and *** represent *p* < 0.01 and *p* < 0.001, respectively. (**B**) A serial dilution of cell numbers was imaged with the fluorescence reader and normalized against the control cells. (**C**) One hour after ICG was added to the OATP1B1-, OATP1B3-expressing and control cells, the cells were monitored under fluorescence microscopy. The ICG was represented in red, whereas cell nucleus were stained by DAPI (blue). C, control; 1B1, OATP1B1-expressing cells; 1B3, OATP1B3-expressing cells. A.U., Absolute Unit.

The ICG intensity of the OATP1B1-expressing cells was approximately 1.5 times greater than that of untransduced cells. Although the ICG uptake efficiency of the OATP1B1-expressing cells was less than that of OATP1B3-expressing cells (Figure 6A), the expression level of OATP1B1 and OATP1B3 are not adjusted to the same level, making the comparison not significant. To further investigate the sensitivity of ICG detection, the detection was performed on a serial dilution of the cells, and the results are normalized with the cell-free group. The results demonstrated that fluorescence detection sensitivity was highest for the OATP1B3-expressing cells. The OATP1B1-expressing cells also had considerable sensitivity to the ICG uptake level. As evident in fluorescence microscopy (Figure 6C), both OATP1B1- and OATP1B3-expressing cells have fluorescent signals intracellularly. Our results suggested that OATP1B1 is a reporter for ICG imaging systems.

## 3. Discussion

In this study, we investigated cellular transportation mechanisms of Gd-BOPTA, which is a widely used contrast agent for clinical MRI. We discovered that Gd-BOPTA could be transported into cells efficiently by OATP1B1 and OATP1B3. This is the first report to propose using OATP1B1 as a Gd-BOPTA reporter gene. Moreover, OATP1B1 was determined to be a better reporter of Gd-BOPTA than OATP1B3 in our study. One study reported that *Slco1a/1b* knockout mice, which had decreased expression of OATP1A1 and OATP1B2, had significant reductions in the uptake of Gd-EOB-DTPA and Gd-BOPTA in the liver [19]. This finding suggested that OATP1A1 and OATP1B2 are primary contributors to the hepatobiliary influx of Gd-BOPTA in mice. Based on sequence information, mouse OATP1B2 is orthologous to human OATP1B1 and OATP1B3 (approximately 70% shared identity), whereas mouse OATP1A1 is orthologous to human OATP1A2, which is expressed in the brain [20]. A subsequent study indicated that the uptake deficit of liver-specific MRI contrast agents in *Slco1a/1b* knockout mice could be rescued by knock-in of human OATP1B1/1B3 [17]. The different enhancement levels between Gd-EOB-DTPA and Gd-BOPTA in OATP1B1/1B3 knock-in mice may be due to the expression ratio of OATP1B1 and OATP1B3, as evidenced in our observation that OATP1B1 is a better transporter for Gd-BOPTA than OATP1B3 is. However, these results suggest that the knock-in mouse might be a powerful model for screening novel MRI contrast agents. Therefore, we intend to investigate an OATP1B1-expressing tumor-bearing nude mouse model in the future to evaluate an OATP1B1 reporter system for use in studying tumor biology and in biomedicine.

Several OATP family—contrast agent combinations have been proposed to be efficacious reporters—for example, ^99m^Tc-mebrofenin hepatobiliary scintigraphy (HBS). In a study of Chinese hamster ovary cells, OATP1BI was determined to be more efficient in transporting ^99m^Tc-mebrofenin than OATP1B3 was. By contrast, OATP1B3 is more capable of ICG transportation than are OATP1B1 and OATP2B1 [21]. These findings highlight the divergent substrate preferences of transport proteins. Accordingly, further identifying the specific human transporter proteins for various imaging reagents is an important goal in promoting the development of molecular imaging technology. Consequently, a high-throughput method to facilitate reporter gene discovery should be developed. Although several high-throughput screening methods for imaging compounds and reporters have been proposed [22,23,24], a platform specifically for high-throughput MRI contrast agent screening has not yet been developed. Our MR reporter evaluation system that can scan 96 well samples in a single session may represent a high-throughput MR scanning platform that can facilitate the discovery of MR reporters.

Protein–protein interactions may play a crucial role in the functions of transporter proteins, such as the organic anion transporter OAT1 [25,26,27,28] and ATP-binding cassette transporter [29]. Some transporter proteins have been demonstrated to form homo- or hetero-oligomers that lead to altered substrate transportation efficiency. OATP1B1 has a relatively high affinity with estrone-3-sulfate when it forms an oligomer mutation of particular amino acids with a GxxxG motif, which is associated with membrane protein dimerization (Figure 1) [30,31] and affects its oligomerization and activity [32]. Moreover, OATP1B3 can form homo-oligomers and hetero-oligomerize with OATP1B1, NTCP, and OCT1 [33]. Coexpression of OATP1B3 with OATP1B1, NTPC, or OCT1 affects its plasma membrane expression and transport kinetics [34]. However, the capabilities of a single transporter serving as an imaging reporter gene might be limited because of substrate preference. Future evaluation of co-expressing transporters could further expand applications.

Studies have found a relationship between OATP1B1 and OATP1B3 and cancers. The mRNA of OATP1B1 was detected in the colon, prostate, lung, and pancreas of patients with cancer [35,36]. OATP1B3 was reported to be expressed in breast, gastrointestinal tract, lung, prostate, and pancreatic cancers [37,38,39,40,41,42]. This cancer-type OATP1B3 (ct-OATP1B3), mainly located in the cytoplasm, might have a low transport capability compared with normal OATP1B3 due to a lack of N-terminal membrane-trafficking peptides (Figure 1) [43]. One study showed that ct-OATP1B3 has poor transport efficiency for CCK-8 in colon and pancreatic cancer cell lines [44]. By contrast, it has been reported that ct-OATP1B3 efficiently transports its specific substrates, including Gd-EOB-DTPA, and is properly located on the plasma membrane in 293T cells [45].

Overexpression of wild-type OATP1B3 provides significant apoptotic resistance for colon cancer cells by affecting the p53-dependent pathway, but this result was not observed for transport-defected mutant OATP1B3 [39]. Moreover, the expression level of ct-OATP1B3 is upregulated in hypoxic environments [46,47], which is a characteristic feature of solid cancers [48,49]. These results imply that the expression of ct-OATP1B3 plays a role in the biological functions of tumors. Therefore, the divergent substrate specificity of OATPs may serve as a drug-selection platform where the aim is to inhibit the noncanonical cytoplasmic function of ct-OATP1B3. Cancer-specific transcript mRNA is an excellent target for the development of cancer therapies and biomarkers. Previous work successfully utilized herpes simplex virus-1 thymidine kinase with *trans*-splicing towards ct-OATP1B3 to develop a cancer suicide gene therapy in vitro and in vivo [50]. However, the transportation capability of ct-OATP1B3 for ICG and Gd-EOB-DTPA and the correlations between ct-OATP1B3 and cancer prognosis are still unknown. Our study identified a valuable contrast agent candidate, Gd-BOPTA, that could be added to the screening pipeline of the transportability assay of ct-OATP1B3. Whether ct-OATP1B3 represents a reporter candidate or a prognostic indicator for cancer treatment outcomes is of clinical significance, with applications for noninvasive cancer diagnosis and outcome prediction.

OATP1B1 was also detected in tumor tissues. Investigation whether OATP1B1 exists as a unique cancer-type isoform is crucial. Moreover, the OATP1B1 T521C polymorphism has been identified as an independent prognostic indicator for breast cancer [51]. The association of the transporting capability of ICG, Gd-BOPTA, or Gd-EOB-DTPA with the OATP1B1 T521C polymorphism is not well understood. The differential transporting capability of OATP1B1 T521C might be useful for in vivo molecular imaging for personalized medicine targeting this polymorphism.

Despite the highly conserved sequence homology between OATP1B1 and OATP1B3, our results indicated divergent substrate preferences for Gd-based contrast agents within the OATP family. We also demonstrated that even the simple modification of adding the N-terminal FLAG tag to OATP1B1-expressing 293T cells affected Gd-BOPTA uptake capability. Moreover, our data demonstrated different ICG transporting abilities between OATP1B1- and OATP1B3-expressing cells. Genetically engineered point-mutation OATP1B1, OAPT1B3, or even ct-OATP1B3 could be the subject of further research into optimizing MR and optical dual-imaging reporters.

## 4. Materials and Methods

### 4.1. Cell Line and Culture

HT-1080 fibrosarcoma cells and HEK293T cells were obtained from Taiwan Bioresource Collection and Research Center (BCRC, HsinChu, Taiwan) and cultured in Dulbecco’s modified Eagle’s medium (Thermo Fisher Scientific, Waltham, MA, USA) supplemented with 10% fetal bovine serum (Biologic Industries, Cromwell, CT, USA), 100 U/mL penicillin, and 100 mg/mL streptomycin (Thermo Fisher Scientific, Waltham, MA, USA). These cells were cultured in an incubator with a humidified atmosphere containing 5% CO_2_ at 37 °C. We passaged the cells at 70–80% confluence.

### 4.2. Vector Construction and Cell Transfection and Transduction

The plasmid pWPXL-OATP1B3-ires-Puro was constructed previously [14]. The lentiviral vector pLEX-MCS and the mammalian expression vector pFLAG-CMV were generous gifts from Ming-Jium Shieh at National Taiwan University. The open reading frame of *SLCO1B1* was amplified from human cDNA and then cloned into *Spe*I/*Mlu*I sites of pLEX-MCS or *PstI/EcoRV* sites of pFLAG-CMV. The final construct was named pLEX-1B1 and pFLAG-1B1. The sequencing results are shown in Supplementary Figure S1. Transient transfection was performed by PolyJet™ In Vitro DNA Transfection Reagent (Signagen, Frederick, USA) and the protocol was followed manufacturer document. Briefly, 1 × 10^6^ 293T cells were seeded on 10 cm^2^ dish, medium was renewed 3 h before transfection, and for each sample, diluted 1 μg plasmid DNA in PolyJet™ reagent (Signagen, Frederick, USA) and drop-wise the mixture onto the medium. Subsequent assays were carried out after transfection 48 h. Lentivirus was produced according to the protocol in *Nature Protocols* [52]. Briefly, 2 × 10^5^ HT-1080 cells were plated in 6-well plates for 24 h. Later, these HT-1080 cells were transduced at the multiplicity of infection of 5. At 24 h after infection, cell selection started with 2 ug/mL puromycin (Milliore-Sigma, St. Louis, USA,) for 10 days. This puromycin selection medium was changed every 2 days.

### 4.3. Western Blotting

Cell lysis was performed using RIPA buffer (Roche, Mannhein, Germany). The concentration of total protein extract was measured by using a Pierce BCA Protein Assay Kit (Thermo Fisher Scientific). Each lane of SDS-PAGE gel was loaded with 30 μg of protein extract and electrotransferred onto nitrocellulose membranes (Sartorius, Göttingen, Germany). The blots were blocked using 5% nonfat milk and 1% bovine serum albumin (BSA, Biologic Industries, Cromwell, CT, USA) in tris-buffered saline with Tween, incubated separately with primary antibodies against OATP1B1 or OATP1B3 (1:1000, Abcam, Cambridge, UK) and β-actin (1:5000; Thermo Fisher Scientific) at 4 °C overnight, and then were incubated with 1:5000 horseradish peroxidase (HRP)-conjugated rabbit/mouse anti-IgG for 1 h at room temperature. Protein signals were detected by using the Immobilon Western Chemiluminescent HRP Substrate (Millipore-Sigma) with a BioSpectrum 810 Imaging System (UVP, Upland, CA, USA).

### 4.4. Immunofluorescence

Cells were seeded on 8-well chamber slides and incubated overnight. They were fixed in 4% formaldehyde (Millipore-Sigma, St. Louis, USA) overnight, and then were blocked with 5% BSA. Samples were incubated with 1:100 rabbit polyclonal anti-OATP1B1 antibody and 1:100 anti-OATP1B3 antibody (Thermo Fisher Scientific, Waltham, MA, USA) at 4 °C overnight. After washing in phosphate-buffered saline (PBS) three times, the slides were treated with 488-conjugated goat antirabbit IgG antibodies (Thermo Fisher Scientific, Waltham, MA, USA) and counterstained with DAPI at room temperature for 1 h. These cover slides were scanned using a Fluorescent Cell Imager (ZOE, Bio-Rad, Hercules, CA, USA).

### 4.5. In Vitro MRI Contrast Agents Uptake Assay

A 10 cm dish was seeded with 1 × 10^7^ cells overnight and treated with either 2 mM Gd-EOB-DTPA (BayerPharma, Berlin, Germany) or 2 mM Gd-BOPTA (GE Healthcare, Waukesha, WI, USA) for 4 h. Some cells were treated with contrast agents at 4 °C as a negative control. Cells were trypsinized an St. Louis, USA d then washed three times with cold PBS. After the cells were centrifuged for 5 min at 1500 rpm in 0.2-mL tubes at 4 °C, they were detected using a clinical 1.5-T MRI system (MAGNETOM Aera, Siemens Healthineers). The cells were harvested and placed in a homemade water tank. The tank was then placed in an 8-channel head coil for imaging. Two-dimensional T1-weighted fast spin-echo pulse sequences were used (TR/TE = 700/20 ms). The slice thickness was 1.5 mm with a 0.5 mm gap, and the field of view was 20 cm × 14.5 cm in the transverse plane and 10 cm × 4 cm in the sagittal plane. The images were processed using *syngo* fastView (version VX57M, Siemens Healthineers) and were then analyzed with ImageJ software (NIH, Bathesda, USA) [53].

### 4.6. Detection of the Cellular Uptake of ICG

The ICG intensity detection was done by using the immunofluorescence reader. Briefly, 1 × 10^5^ cells were serially diluted and seeded in 96-well black plates and treated with 50 μg/mL ICG for 1 h. Excess ICG was removed by washing three times with PBS. All data were acquired under similar parameters (excitation/emission wavelength: 780/830 nm; shaking duration: 2 s) and then normalized with a control group or cell-free group.

## 5. Conclusions

We verified the transporting capability of both OATP1B1 and OATP1B3 for Gd-BOPTA in permanent cell line transduction models that cellular MRI has demonstrated. Moreover, both OATP1B1 and OATP1B3 can take up ICG evidenced by fluorescent microscopy, and therefore, OATP1B1 and OATP1B3 are both effective dual-imaging reporters. These findings have the potential to further research in developing novel MR reporting systems. As the rising role of cancer-type OATP1B3 and the potential role of OATP1B1 in cancer prognosis prediction, evaluation outcomes of cancers in which OATPs are overexpressed might be possible by either MRI or fluorescence imaging. The correlation of cancer-type OATP1B3 and its imaging capability as well as the identification of role of cancer-type OATP1B1 should be pursued.

## Data Availability

The data presented in this study are available on request from the corresponding author.

## Supplementary Materials

The following are available online at https://www.mdpi.com/article/10.3390/ijms22168797/s1. Figure S1: The sequence result of plasmid construct pLEX-1B1 and pFLAG-1B1.
