# Supplementary material for: Organic Anion Transporting Polypeptide 1B1 Is a Potential Reporter for Dual MR and Optical Imaging
Source: Int J Mol Sci. 2021 Aug 16;22(16):8797. doi: 10.3390/ijms22168797 (PMC8395777; doi:10.3390/ijms22168797)
Supplement: Supplementary file 1 [file ijms-22-08797-s001.zip › Supplementary-Methods and materials.pdf]

For DNA sequencing, both plasmids were sent to MISSION BIOTECH Company (Taipei, Taiwan). plasmid DNA including pLEX-1B1 and pFLAG-1B1 at the amount of 100 ng of for each sample was amplified and Sanger sequenced. The primer was used pLEX-MCS-F for forward sequencing, and pLEX-MCS-R for reverse sequencing. The information of sequencing primers were listed below.

Primer information

Primer Name	Length	Tm	%GC	Sequence 5’-3’
pLEX-MCS-F	20	59.83	45	CACCAAAATCAACGGGACTT
pLEX-MCS-R	24	60.3	50	ATATAGACAAACGCACACCGGCCT
