# Supplementary figures and images for: Organic Anion Transporting Polypeptide 1B1 Is a Potential Reporter for Dual MR and Optical Imaging
Source: Int J Mol Sci. 2021 Aug 16;22(16):8797. doi: 10.3390/ijms22168797 (PMC8395777; doi:10.3390/ijms22168797)

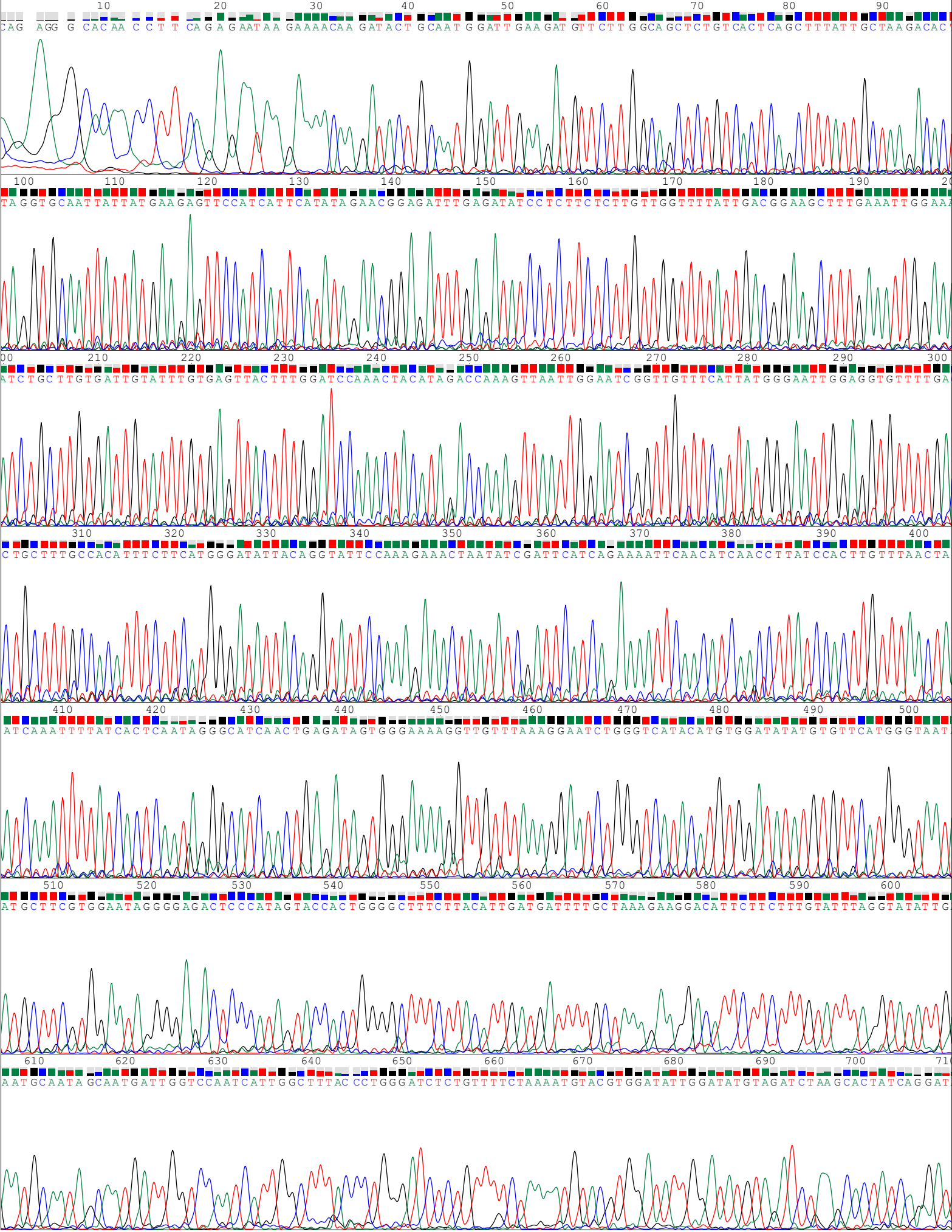

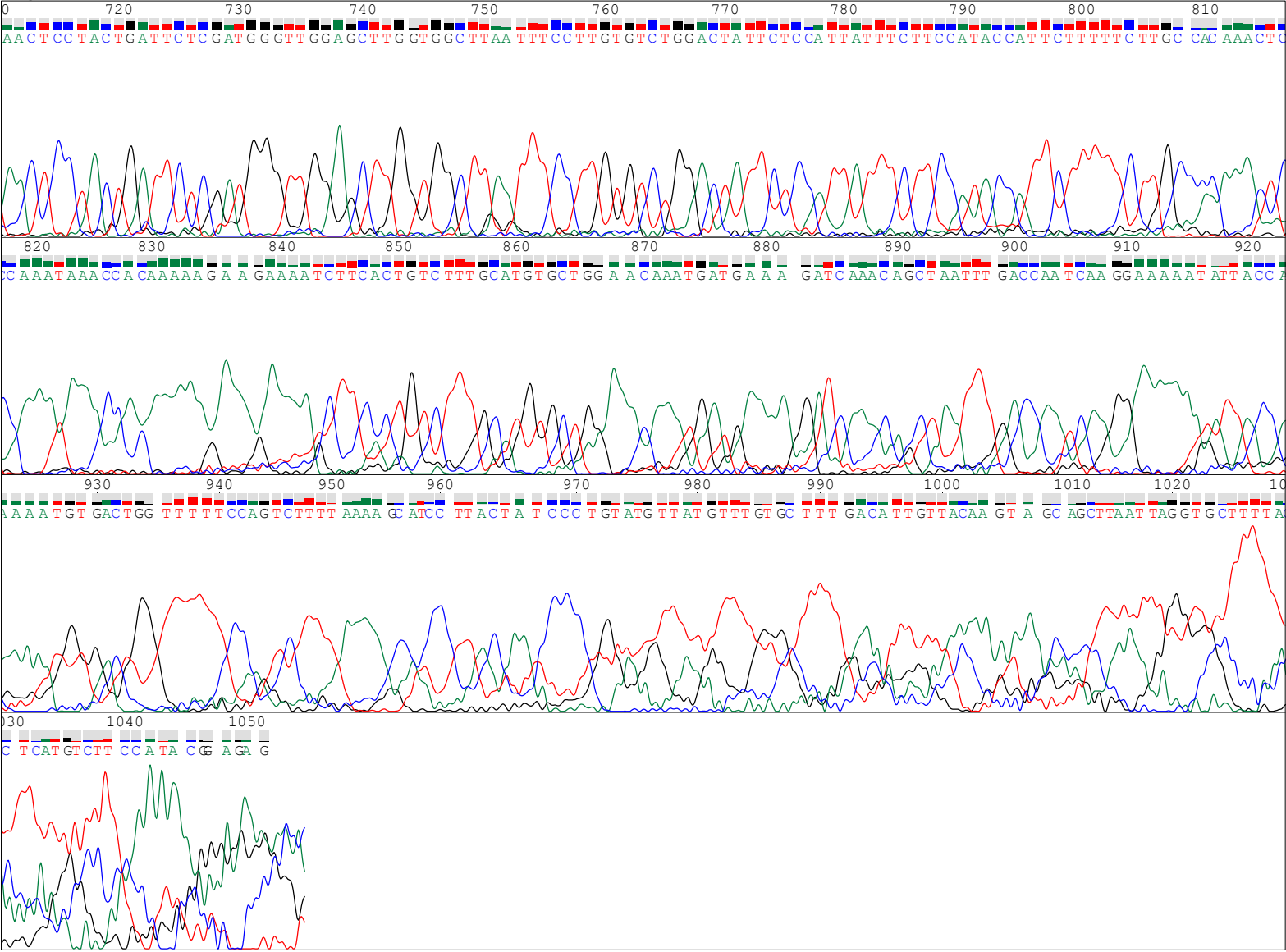

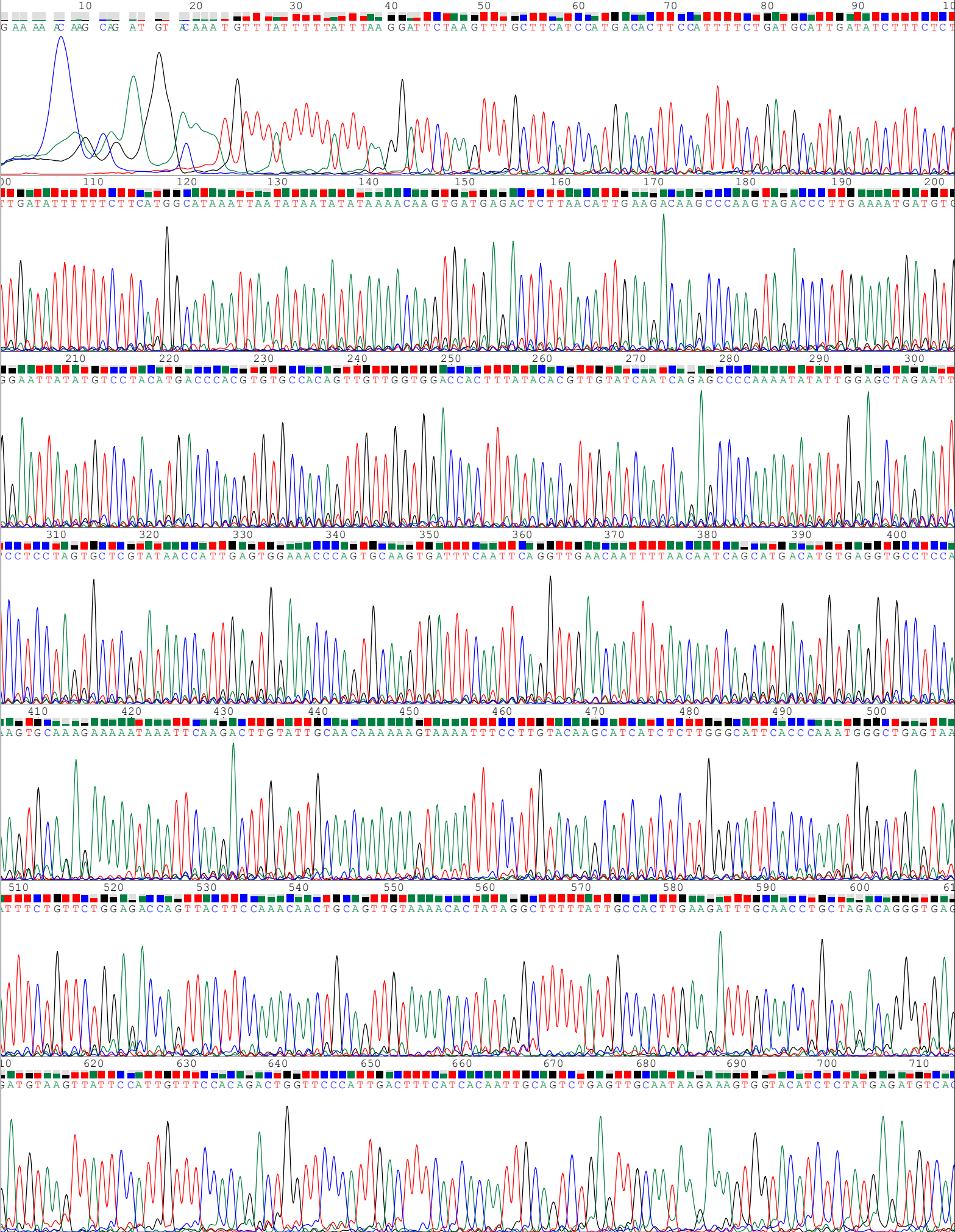

Supplement: Supplementary file 1 [file ijms-22-08797-s001.zip › S1.pdf]
